# Transgenic mice overexpressing glia maturation factor-β, an oxidative stress inducible gene, show premature aging due to Zmpste24 down-regulation

**DOI:** 10.18632/aging.100779

**Published:** 2015-07-13

**Authors:** Rika Imai, Kanae Asai, Jun-ichi Hanai, Masaru Takenaka

**Affiliations:** ^1^ Clinical Nutrition and Internal Medicine, Kobe Women's University, Kobe 654–8585, Japan; ^2^ Renal Division & Division of Interdisciplinary Medicine and Biotechnology, Beth Israel Deaconess Medical Center, Harvard Medical School, Boston, MA 02215, USA

**Keywords:** glia maturation factor-β, prelamin A, Zmpste24, aging, oxidative stress

## Abstract

Glia Maturation Factor-β (GMF), a brain specific protein, is induced by proteinuria in renal tubules. Ectopic GMF overexpression causes apoptosis *in vitro* via cellular vulnerability to oxidative stress. In order to examine the roles of GMF in non-brain tissue, we constructed transgenic mice overexpressing GMF (GMF-TG). The GMF-TG mice exhibited appearance phenotypes associated with premature aging. The GMF-TG mice also demonstrated short lifespans and reduced hair regrowth, suggesting an accelerated aging process. The production of an abnormal lamin A, a nuclear envelope protein, plays a causal role in both normal aging and accelerated aging diseases, known as laminopathies. Importantly, we identified the abnormal lamin A (prelamin A), accompanied by a down-regulation of a lamin A processing enzyme (Zmpste24) in the kidney of the GMF-TG mice. The GMF-TG mice showed accelerated aging in the kidney, compared with wild-type mice, showing increased *TGF-β1*, *CTGF* gene and serum creatinine. The gene expression of p21/waf1 was increased at an earlier stage of life, at 10 weeks, which was in turn down-regulated at a later stage, at 60 weeks. In conclusion, we propose that GMF-TG mice might be a novel mouse model of accelerated aging, due to the abnormal lamin A.

## INTRODUCTION

Chronic diseases, including cardiovascular disease, diabetes and cancer, account for a high rate of mortality [1], and aging is a notable risk factor for the progression of chronic diseases [2, 3]. The prevalence of chronic kidney disease (CKD) increases with age [4], and CKD accelerates the progression of atherosclerosis and increases propensity to oxidative stress [5]. Accordingly, CKD is a significant risk factor for cardiovascular disease, as well as for the progression to end-stage renal disease [4]. Thus, CKD has an important impact on human health and longevity.

Chronic proteinuria is not only a sign of CKD, it also plays an important role in the progression of CKD [6]. Previously, it has been shown that proteinuria induces the ectopic expression of a brain specific protein, glia maturation factor-β (GMF), in renal proximal tubular cells [7, 8]. GMF was initially identified in the nervous system, and thought to play a role as a growth/differen-tiation factor and as an intracellular regulator of stress-related signals [9, 10]. It has been also suggested that GMF overexpression in brain tissue might be implicated in the progression of neurodegenerative diseases, such as Alzheimer's disease, Parkinson's disease and multiple sclerosis [10–13]. GMF overexpression in non-brain cells causes a vulnerability to oxidative stress *in vitro*, resulting in apoptosis [14]. This function was also confirmed by evidence showing that GMF-null astrocytes increase resistance to oxidative stress [15]. In this study, we constructed transgenic mice overexpressing GMF (GMF-TG), and for the first time, revealed the roles of GMF overexpression in non-brain tissue *in vivo*.

Hutchinson-Gilford progeria syndrome (HGPS) is a genetic accelerated aging disease caused by the alteration of the lamin A protein and their assembly [16]. Lamin A is processed from prelamin A, through a cleavage by Zmpste24, a zinc-metalloproteinase [16]. Mutations of lamin A/C (*Lmna*) gene or the *Zmpste24* gene produce abnormal lamins, such as progerin or prelamin A, respectively [16], which can cause disassembly of the nuclear envelope proteins, subsequently accompanied by accelerated aging due to laminopathies [16]. Laminopathies can be further classified into primary and secondary laminopathies [16]. The primary laminopathies are due to mutations in the *Lmna* gene and secondary laminopathies are caused by mutations in the *Zmpste24* gene [16]. Although the clinical features of laminopathies are radical, the abnormalities associated with lamin A are normally observed in healthy human aging, suggesting that the accumulation of abnormal lamin A protein is associated with organismal physiological aging [17–20].

In this study, we first found premature-aging phenotypes, such as alopecia and skin atrophy, in the appearance of the GMF-TG mice. Because these phenotypes seemed to be similar to those of laminopathy-based premature aging, we investigated the state of the lamin A. We identified an accumulation of abnormal lamin A (prelamin A), accompanied by a reduction in the Zmpste24 expression in the GMF-TG mice, suggesting that accelerated aging phenotypes in the GMF-TG mice might be associated with this laminopathy, caused by prelamin A accumulation. In regards to the mechanisms of the laminopathy-based premature aging in the GMF-TG mice, we consider it possible that GMF over-expression in non-brain tissue may cause secondary laminopathy through enhancing oxidative injuries, probably due to its vulnerability to oxidative stress [14]. This proposition suggests the novel role of GMF overexpression in non-brain tissue *in vivo*. Thus, the results of this study contribute to our understanding of the connection between aging, age-related disease and various chronic conditions, and provide new insight into the accumulation of prelamin A, which is not only caused by genetic mutation, but also by stress signals, such as oxidative stress.

## RESULTS

### Transgenic mice overexpressing GMF

**Figure 1:**
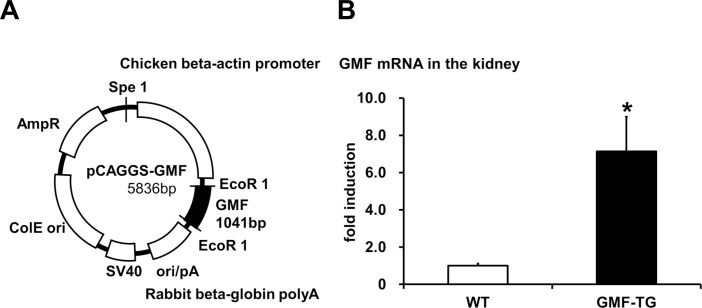
Preparation of transgenic mice overexpressing GMF (GMF-TG) (**A**) This figure shows the construct used to prepare the transgenic mice. The construct was prepared by cloning the coding region of GMF (97–915 bp) in a pCAGGS vector. (**B**) This figure shows that the gene expression of GMF in the kidney of the GMF-TG mice was significantly higher (about 7-fold) than that of the wild-type (WT) mice. The data is shown as means ± S.E. (WT; n=3, GMF-TG; n=3). *; *P* < 0.05 vs. WT mice.

It is considered that GMF is normally expressed in the brain in a tissue-specific manner [9, 10]. However, GMF is also induced ectopically in renal proximal tubules by proteinuria [7, 8]. In order to analyze the roles of a previously unknown GMF overexpression in non-brain tissue, we first created transgenic mice overexpressing GMF (GMF-TG mice), as described in the ‘Materials and Methods’ section (Figure 1A).

We confirmed that the specific sequence, which was incorporated in a genome, was expressed in the correct direction in established transgenic lines (data not shown). Next, we conducted quantitative PCR analyses to confirm the expression of GMF in the GMF-TG mice. In this study, we used kidney tissue, because it has been shown that GMF overexpression is ectopically induced in kidney tissue by proteinuria [7, 8]. The results of the quantitative PCR analyses, employing mRNA obtained from the kidneys of wild-type and GMF-TG mice, showed that GMF mRNA from the GMF-TG mice increased significantly (approximately 7-fold), compared with the wild-type mice (Figure 1B).

### Phenotypes of GMF-TG mice

**Figure 2:**
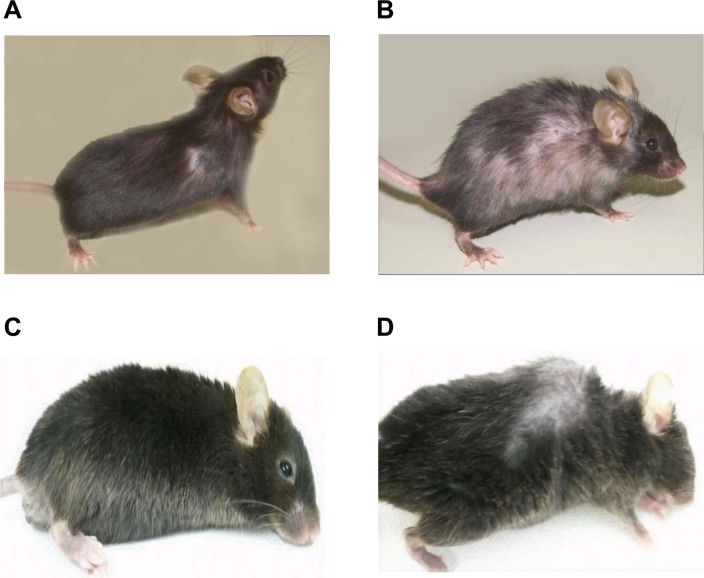
Photograph of WT and GMF-TG mice (**A**-**B**) These photographs show the representative appearance of WT (**A**) and GMF-TG (**B**) mice at 80 weeks, respectively. The GMF-TG mice showed alopecia and skin atrophy. These phenotypes were not detected in the aged-matched wild-type mice. (**C**-**D**) Indicators of aging phenotypes, such as spinal curvature, were detected in the GMF-TG (**D**) mice, but not in the WT (**C**) mice at 110 weeks.

**Figure 3:**
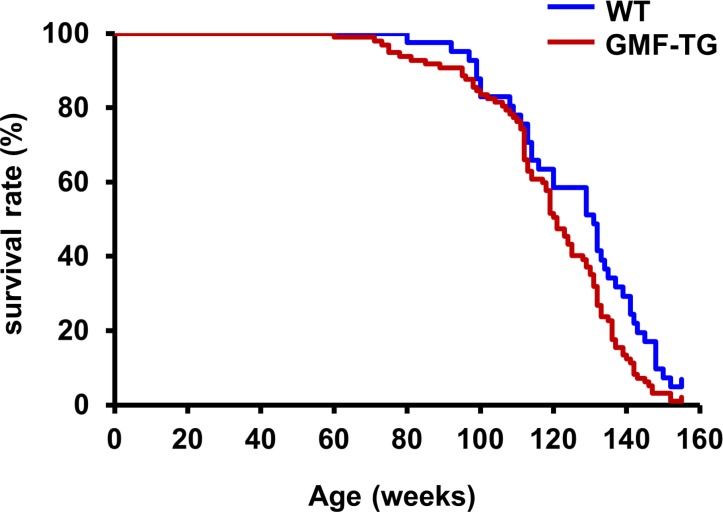
Kaplan-Meier graph of WT and GMF-TG mice This figure shows a Kaplan-Meier representation of the survival curves, which revealed that the GMF-TG mice died significantly earlier than the WT mice. The GMF-TG mouse and the two wild-type mice were alive when the data analysis was performed. None of the mice exhibited any signs of distress or pain due to the clinical symptoms used to assess health and welfare. (WT; n=41, GMF-TG; n=97). *P < 0.05 vs. WT mice.

We bred GMF-TG mice with wild-type mice. During the breeding period, only the GMF-TG mice began to show signs of aging in appearance [21], including hair graying and lack of hair glossiness, at about the age of 30 weeks (hereafter, 30 weeks, etc.). It is generally recognized that oxidative stress is one of the major factors that promote the aging process in organisms [22, 23]. We hypothesized that GMF overexpression in non-brain tissue resulted in accelerated aging, probably through cellular vulnerability to oxidative stress [14]. Prematurely aged mice exhibit early aging-like appearance phenotypes, including increased hair loss, lordokyphosis of the spine, a shortened lifespan and growth retardation, compared to wild-type mice [24–28]. We monitored the aging-related phenotypes of the GMF-TG and wild-type mice during an experimental period of 155 weeks. The GMF-TG mice developed alopecia early, by about 75 weeks, while the wild-type mice started to show alopecia after about 100 weeks (Figure 2A-B and Supplemental Table 1). Some GMF-TG mice also exhibited skin atrophy and spinal curvature. These phenotypes were not detected in the wild-type mice (Figure 2 and Supplemental Table 1). We also found that some of the GMF-TG mice died within 60 weeks. The average lifespan of the GMF-TG mice was about 119 weeks, and that of the wild-type mice was about 126 weeks. Kaplan-Meier representations of the survival curves demonstrated that the GMF-TG mice died significantly earlier than the wild-type mice (Figure 3). There was no statistically significant difference in the body weight and size in mature-adult mice (at about 20 weeks) (data not shown).

**Figure 4:**
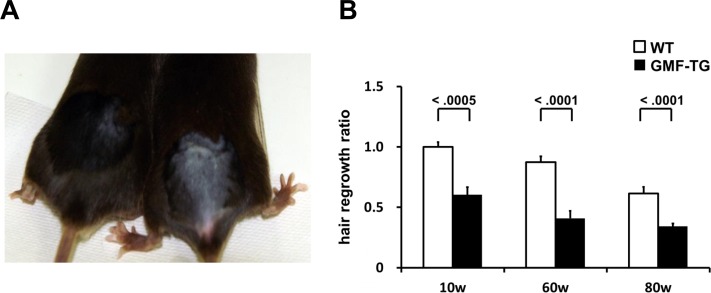
Hair regrowth phenotypes in WT and GMF-TG mice (**A**) This photograph shows the representative appearance of the WT (Left) and GMF-TG (Right) mice at 10 weeks, 15 days after shaving. Almost no hair regrowth was observed in the GMF-TG mice, whereas the WT mice displayed robust hair regrowth. (**B**) The figure shows the results for the hair regrowth ratio in the WT and GMF-TG mice at 10, 60 and 80 weeks, 15 days after shaving. The hair regrowth ratio for the GMF-TG mice declined significantly, compared with the WT mice at the same age. The mean of the hair regrowth results for the WT mice is shown as 1. The data is shown as means ± S.E. (10, 60, 80w WT; n=3, 10w GMF-TG; n=6, 60, 80w GMF-TG; n=5).

**Figure 5:**
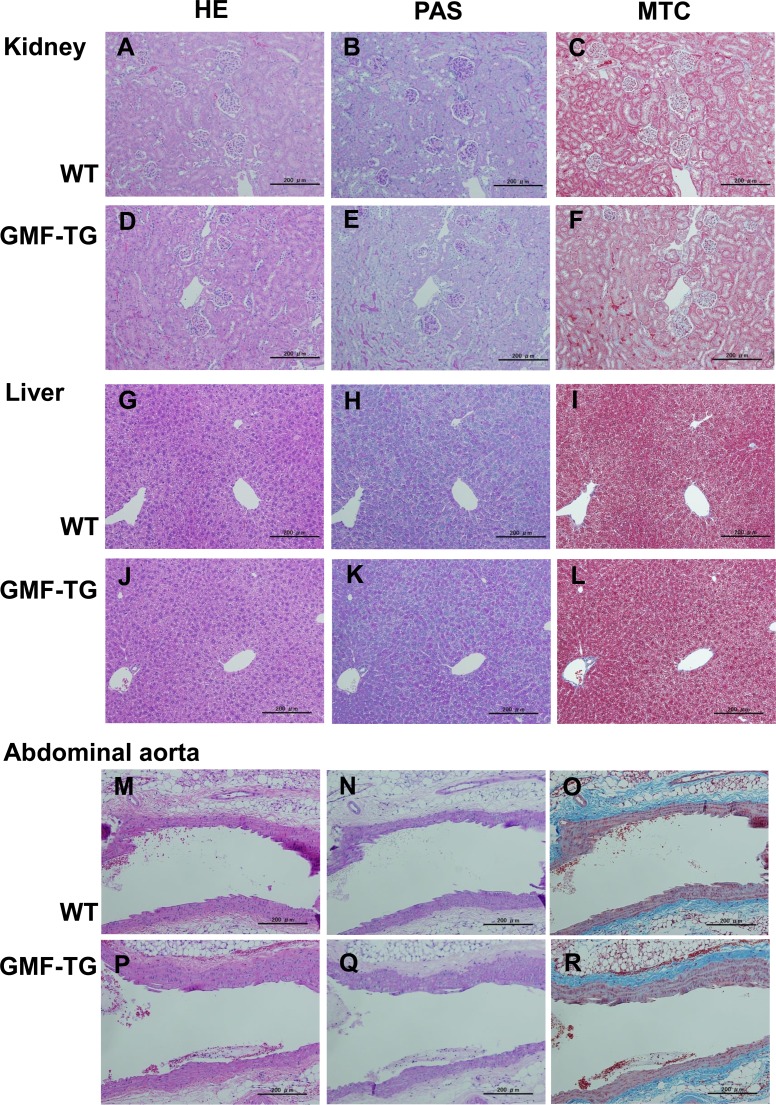
Histological appearance of the kidney, liver and abdominal aorta in WT and GMF-TG mice (**A**-**F**) These photographs show an overview of the hematoxylin-eosin (HE) (**A** and **D**), periodic acid–schiff (PAS) (**B** and **E**), and Masson trichrome (MTC) (**C** and **F**) stained kidney sections in the WT (**A**-**C**) and GMF-TG (**D**-**F**) mice at 30 weeks. (**G**-**L**) These photographs show an overview of the HE (**G** and **J**), PAS (**H** and **K**), and MTC (**I** and **L**) stained liver sections in the WT (**G**-**I**) and GMF-TG (**J**-**L**) mice at 30 weeks. (**M**-**R**) These photographs show an overview of the HE (**M** and **P**), PAS (**N** and **Q**), and MTC (**O** and **R**) stained abdominal aorta sections in the WT (**M**-**O**) and GMF-TG (**P**-**R**) mice at 30 weeks. These findings revealed no histological differences between the WT and GMF-TG mice. Magnifications: ×100, Scale Bar = 200 μm.

Next, we examined aging-related changes in the tissue structure. Degenerative changes in skin tissue are readily visible, so they can be detected easily [27]. Decreased hair regrowth has often been reported in prematurely aged mice [25–27]. Because hair growth assays can be employed to monitor degenerative changes without adversely affecting the mice, we employed them to investigate the influence of aging on skin tissue. When dorsal segments of skin were shaved on age-matched mice, the GMF-TG mice showed sparse hair regrowth after 15-days. In contrast, at the same age, the wild-type mice displayed robust hair regrowth (Figure 4A). The hair regrowth ratio significantly declined in the GMF-TG mice at 10, 60 and 80 weeks, compared with the wild-type mice at the same age (Figure 4B). In the kidney, liver, and abdominal aorta at 30 weeks, there were no histologically detectable changes between the GMF-TG and wild-type mice, as indicated in Figure 5. These results suggested that the GMF-TG mice developed mild premature aging phenotypes.

**Figure 6:**
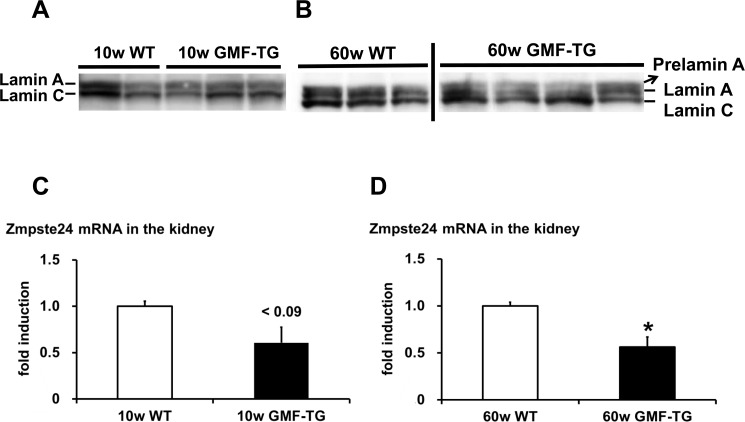
Western blot of lamin A/C and the expression of Zmpste24 in WT and GMF-TG mice (**A**-**B**) These figures show the results from western blot analyses of the lamin A/C protein in the kidney of the WT and GMF-TG mice at 10 and 60 weeks. Prelamin A was absent in the WT mice at 10 and 60 weeks (**A**-**B**; Left) and the GMF-TG mice at 10 weeks (**A**; Right), but it was detectable in the GMF-TG mice at 60 weeks (**B**; Right). There were no significant differences between the lamin C protein levels in the WT and GMF-TG mice, confirming equal loading. (**C**) The expression of Zmpste24 mRNA tended to decrease in the kidneys of the GMF-TG mice at 10 weeks, compared with the WT mice. The data is shown as means ± S.E. (10w WT; n=3, 10w GMF-TG; n=3). *P* < 0.09 vs. 10w WT mice. (**D**) The expression of Zmpste24 mRNA decreased in the kidneys of the GMF-TG mice at 60 weeks, compared with the WT mice. The data is shown as means ± S.E. (60w WT; n=4, 60w GMF-TG; n=4). *; *P* < 0.01 vs. 60w WT mice.

HGPS is associated with premature alopecia, which is one of the well-known premature-aging syndromes due to laminopathies, seen in humans [29]. Cells and tissue from HGPS patients exhibited an accumulation of abnormal lamin A (progerin) [17, 18]. The phenotypes characterized in the skin of the GMF-TG mice, such as alopecia and skin atrophy, seemed to be similar to that of laminopathy-based premature aging. Therefore, we hypothesized that an accumulation of abnormal lamin A resulted in the accelerated aging phenotypes shown in the GMF-TG mice. In order to analyze the abnormalities of the lamin A in the tissue of the GMF-TG mice, we first examined the lamin A protein in the kidney by western blotting, because GMF over-expression was ectopically induced in kidney tissue by proteinuria [7, 8]. At 10 weeks, no lamin A abnormalities were exhibited in the kidney of either the GMF-TG or wild-type mice (Figure 6A and Figure Supplemental 1A). However, at 60 weeks, an accumulation of abnormal lamin A (prelamin A) was detected in the kidneys of the GMF-TG mice, but not in the wild-type mice (Figure 6B and Figure Supplemental 1B). Next, we evaluated the expression levels of the cleaving enzyme of prelamin A (*Zmpste24*) gene in the kidneys by real-time PCR analyses to confirm the mechanism of the accumulated prelamin A. At 10 weeks, the expression of Zmpste24 mRNA tended to decrease in the GMF-TG mice (Figure 6C). At 60 weeks, a significant decrease was demonstrated in the expression of Zmpste24 mRNA in the GMF-TG mice, compared with the wild-type mice at the same age (Figure 6D). These results demonstrated that the GMF-TG mice exhibited an accumulation of prelamin A, accompanied by a reduction of *Zmpste24* gene expression in the kidney tissue. On the basis of these results, we investigated the degree of aging in kidney tissue of the GMF-TG mice. In the kidney, aging-associated changes are characterized by structural changes, including glomerulosclerosis and interstitial fibrosis [30, 31], as well as the decline of renal function [31]. It has been suggested that the transforming growth factor-β1 (*TGF-β1*) gene is one of the factors that promote the process of renal interstitial fibrosis associ ated with aging [30].

**Figure 7:**
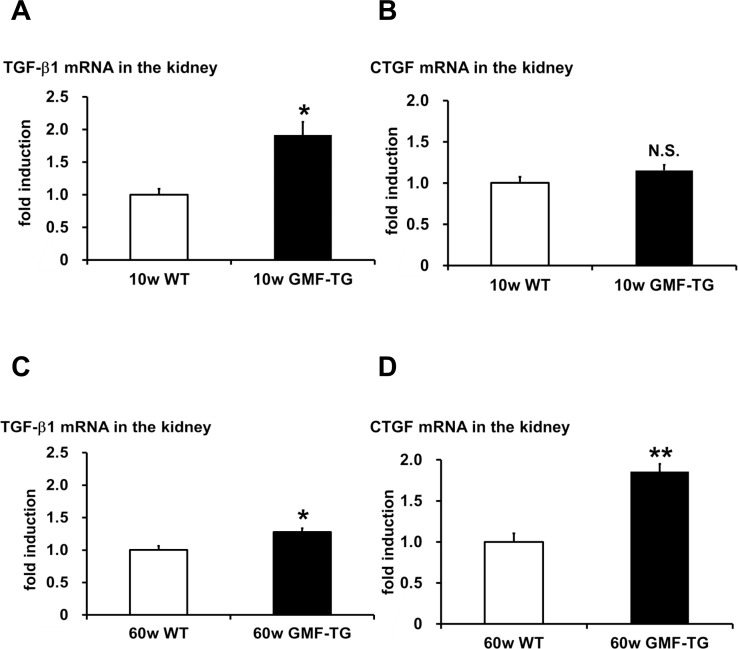
The expression of TGF-β1 and CTGF in WT and GMF-TG mice (**A**-**B**) The expression of transforming growth factor-β1 (TGF-β1) (**A**) mRNA increased in the kidneys of the GMF-TG mice at 10 weeks, compared with the WT mice. However, no significant differences were shown in the expression of connective tissue growing factor (CTGF) (**B**) mRNA in the kidney of the GMF-TG mice at 10 weeks, compared with the WT mice. TGF-β1; The data is shown as means ± S.E. (10w WT; n=4, 10w GMF-TG; n=5). CTGF; The data is shown as means ± S.E. (10w WT; n=3, 10w GMF-TG; n=4). *; *P* < 0.01 vs. 10w WT mice. N.S.; not significant versus 10w WT mice. (**C**-**D**) These figures demonstrated a significant increase in TGF-β1 (**C**) and CTGF (**D**) mRNA in the kidneys of the GMF-TG mice at 60 weeks, compared with the WT mice at the same age. The data is shown as means ± S.E. (60w WT; n=4, 60w GMF-TG; n=4. *; *P* < 0.05 vs. 60w WT mice. **; *P* < 0.001 vs. 60w WT mice.

**Figure 8:**
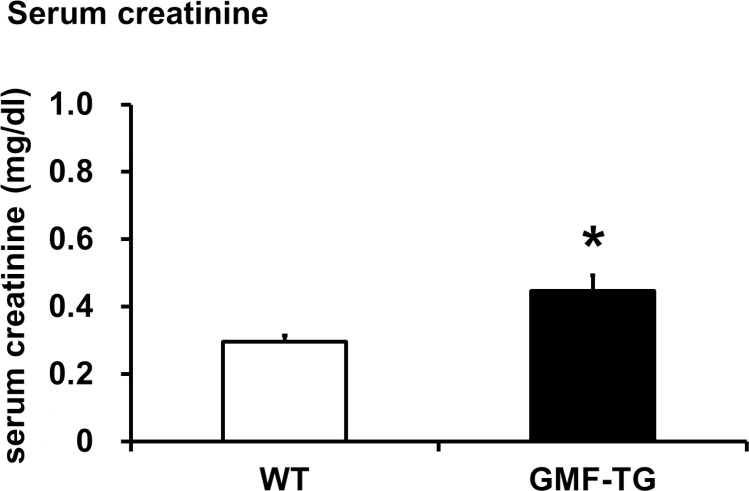
The serum creatinine levels in WT and GMF-TG mice This figure shows the result of the serum creatinine levels in the WT and GMF-TG mice, which revealed that serum creatinine was increased in the GMF-TG mice, compared with the WT mice. The data is shown as means ± S.E. (WT; n=4, average age; 84.8 ± 0.25 weeks, GMF-TG; n=4 average age; 72.8 ± 6.24 weeks). *; *P* < 0.05 vs. WT mice.

The connective tissue growing factor (*CTGF*) gene is known as a downstream mediator of TGF-β1. In order to investigate age-associated changes in the kidneys, we evaluated the expression levels of the *TGF-β1* and *CTGF* genes by real-time PCR analyses. In the kidney tissue of the GMF-TG mice at 10 weeks, the expression of TGF-β1 mRNA increased significantly, compared with the wild-type mice (Figure 7A). However, there was no statistically significant difference between the expression of CTGF mRNA of the kidney of the GMF-TG and wild-type mice at 10 weeks (Figure 7B). Importantly, in the GMF-TG mice at 60 weeks, the expression of both TGF-β1 and CTGF mRNA in the kidney increased significantly, compared with that of the wild-type mice (Figure 7C and D). It has been reported that serum creatinine was increased in old mice compared with young mice, suggesting the decline of renal function with advancing age [31]. Figure 8 showed that serum creatinine was increased in the old GMF-TG mice (Average age: 72.8 weeks) compared with the old wild-type mice (Average age: 84.8 weeks). These results demonstrated that the GMF-TG mice showed premature-aging phenotypes in the kidney tissue, probably through an accumulation of prelamin A. These findings suggested that the GMF-TG mice might show a tendency for laminopathy-based premature aging.

### The mechanisms of laminopathy-based premature aging in the GMF-TG mice

**Figure 9:**
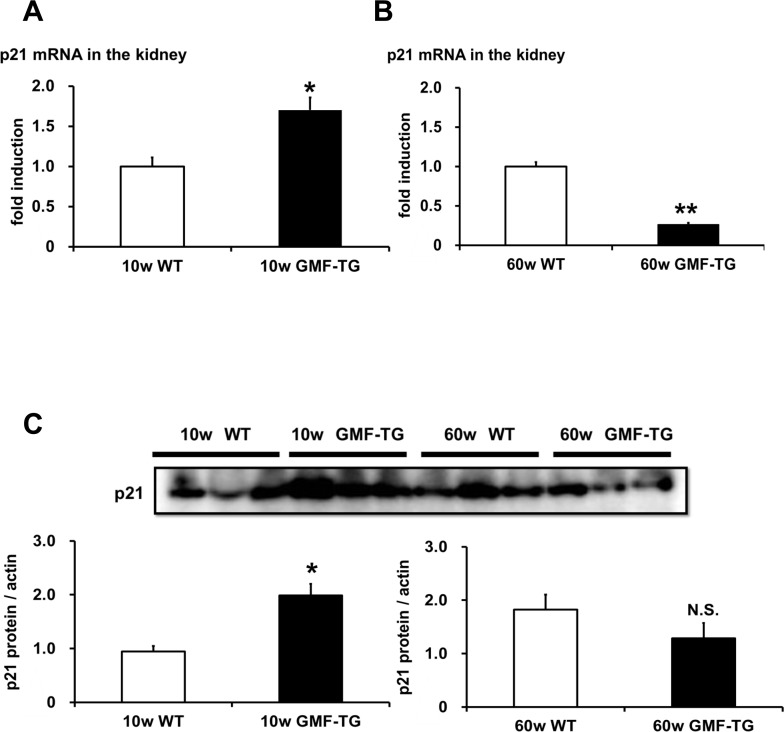
The expression of p21/waf1 mRNA and protein in WT and GMF-TG mice (**A**) The expression of p21/waf1 mRNA increased in the kidneys of the GMF-TG mice at 10 weeks, compared with the WT mice. The data is shown as means ± S.E. (10w WT; n=3, 10w GMF-TG; n=4). *; *P* < 0.05 vs. 10w WT mice. (**B**) In the kidneys at 60 weeks, the expression of p21/waf1 mRNA in the GMF-TG mice was reduced significantly, compared to that of the WT mice at the same age. The data is shown as means ± S.E. (60w WT; n=4, 60w GMF-TG; n=4). **; *P* < 0.01 vs. 60 w WT mice. (**C**) This figure shows the results from western blot analyses of the p21/waf1 protein in the kidney of the WT and GMF-TG mice at 10 and 60 weeks. The data shown is representative data on the estimated ratios of p21/waf1 to α-actin, in the case of equivalent protein loading within a gel. Increased p21/waf1 protein levels were detected in the GMF-TG mice at 10 weeks, compared with the WT mice. However, there was no difference in the levels of p21/waf1 protein between the GMF-TG and WT mice at 60 weeks. The data is shown as means ± S.E. (10, 60w WT; n=3, 10, 60w GMF-TG; n=3). *; *P* < 0.05 vs. 10w WT mice. N.S.; not significant versus 60w WT mice.

We attempted to demonstrate the mechanisms of laminopathy-based premature aging in the GMF-TG mice. It has been suggested that premature aging in laminopathy model mice is linked to p53 pathway activation [32]. The activated p53 pathway induces cell/tissue senescence and eventually leads to accelerated aging [22, 23]. We examined whether the expression of *p21/waf1* gene, a p53 downstream target gene, would increase in the kidneys of the GMF-TG mice at 10 and 60 weeks, and found that the expression of p21/waf1 mRNA and protein at 10 weeks increased significantly, compared with the wild-type mice (Figure 9A, C and Figure Supplemental 2). However, at 60 weeks, a significant decrease in the expression of p21/waf1 mRNA was demonstrated in the GMF-TG mice, compared with the wild-type mice at the same age (Figure 9B). There was no statistically significant difference between protein expression of p21/waf1 in the kidney of the GMF-TG and wild-type mice at 60 weeks (Figure 9C and Figure Supplemental 2). These results suggested that the p53 pathway was activated only at an earlier age in the GMF-TG mice, or that some compensative responses might be activated during a later stage in these mice.

## DISCUSSION

The GMF protein is expressed specifically in the brain, mainly in astrocytes [9, 10]. It is thought that GMF functions as a growth/differentiation factor and as an intracellular regulator of stress-related signal transduction [9, 10]. It is also known that GMF is up-regulated in the central nervous system in neuro-degenerative diseases, such as Alzheimer's disease [12, 13], suggesting that GMF overexpression in brain tissue might play important roles in the pathogenesis of these neurodegenerative diseases [10–13]. Previous studies have demonstrated that the ectopic expression of GMF is induced in renal proximal tubular cells by proteinuria [7, 8]. Kaimori et al. showed that GMF overexpression in non-brain cells caused a cellular vulnerability to oxidative stress *in vitro* [14]. Zaheer et al. also reported that GMF-null astrocytes increased resistance to oxidative stress [15]. These findings suggest that the induction of GMF in renal proximal tubular cells by proteinuria might play a key role in the pathogenesis of renal diseases by enhancing oxidative injuries [14]. However, no reports have been published on *in vivo* studies conducted to examine the roles of GMF overexpression in non-brain tissue. Here, we established a novel line of mice, transgenic mice overexpressing GMF (GMF-TG mice) (Figure 1). We found that the GMF-TG mice exhibited a premature onset of aging-associated symptoms seen in physiological human aging, including a lack of hair glossiness, hair graying, alopecia, skin atrophy, and curvature of the spine (Figure 2), indicating that GMF overexpression in non-brain tissue *in vivo* might play a previously unknown role in the aging process. We investigated the development of accelerated aging phenotypes in the GMF-TG mice. Our findings demonstrated that the GMF-TG mice had short lifespans (Figure 3) and showed premature degenerative changes in skin tissue (e.g., reduced hair regrowth) (Figure 4). However there were no visible age-related histological changes in thekidney, liver, and abdominal aorta (Figure 5).

Mutations or altered post-translational lamin A processing lead to an accumulation of lamin A abnormalities, such as progerin or prelamin A [16]. Because these nuclear intermediate filaments, such as lamin A/C, function as a mesh to protect the nucleus from mechanical stress, the alteration of lamin A protein causes a loss of nuclear stability and integrity, manifesting premature-aging syndromes (e.g., HGPS) [16]. The GMF-TG mice were characterized by changes in appearance, such as alopecia and skin atrophy from an earlier stage of life. In appearance, these phenotypes were similar to those seen in laminopathy-based premature aging, such as HGPS. In this study, we investigated whether or not the accelerated aging phenotypes that were observed in the GMF-TG mice were associated with laminopathies. First, we found that the GMF-TG mice accumulated an abnormal lamin A (prelamin A) with age in the kidney tissue (Figure 6A and B). Second, we demonstrated that the GMF-TG mice showed a reduced expression of the cleaving enzyme of prelamin A (Zmpste24) in the kidney tissue, from an earlier stage of life (Figure 6C and D), suggesting that the GMF-TG mice would probably be affected by secondary laminopathy [16] due to an accumulation of prelamin A, accompanied by a reduction in the expression of Zmpste24. Finally, we confirmed that the GMF-TG mice exhibited premature-aging phenotypes in the kidney tissue (e.g., increased *TGF-β1* and *CTGF* genes and serum creatinine) (Figures 7, 8). The results of the present study suggest that the GMF-TG mice might develop ac-celerated aging phenotypes due to secondary laminopathy.

As animal models employed in studies on secondary laminopathies, Zmpste24-knockout mice (Zmpste24−/− mice) are well-known [33]. Zmpste24−/− mice exclusively produce prelamin A as a consequence of Zmpste24 deficiency [33]. These mice exhibit severe phenotypes consistent with human laminopathies (e.g. HGPS), including growth retardation, jaw and bone abnormalities, alopecia and shorter lifespan [33]. Intriguingly, the GMF-TG mice exhibited mild phenotypes without the severe ones, such as growth retardation or extremely short lifespan during the study (Figure 2, 3 and Supplemental Table 1). In regard to the contribution of prelamin A accumulation to the patho-logical aging phenotypes, several studies were conducted using Zmpste24−/− mice and Zmpste24−/− Lmna+/− mice [32, 34]. The accumulation levels of prelamin A of the Zmpste24−/− Lmna+/− mice were significantly reduced, compared to that of Zmpste24−/− mice [32, 34]. In response to this prelamin A reduction, the phenotypes observed in the Zmpste24−/− mice were largely rescued in the Zmpste24−/− Lmna+/− mice [32, 34]. Moreover, there were no differences shown in the body size, weight or lifespan between the Zmpste24−/− Lmna+/− mice and the wild-type mice during the studies [32]. These results suggested that the levels of accumulation of prelamin A might at least partially contribute to the phenotypes seen in the Zmpste24−/− mice, but that is not only the critical determinant for the aging phenotype and the precise underlying pathogenic mechanisms still need to be explored [32, 34]. We detected an accumulation of prelamin A only in the old GMF-TG mice (Figure 6A and B).

The young GMF-TG mice tended to show a reduced expression of the *Zmpste24* gene (Figure 6C). It is conceivable that the amount of prelamin A in the GMF-TG mice might be less than that found in the mouse models employed for secondary laminopathies. Here, we propose that the accumulation levels of prelamin A, accompanied by a reduction in the levels of the *Zmpste24* gene, might be characteristic of the mild phenotypes shown in the GMF-TG mice.

Several studies showed that the presence of lamin A abnormalities, such as progerin or prelamin A, was detected in normal human aging [17–20]. Furthermore, an accumulation of prelamin A in healthy aging was associated with the down-regulation of Zmpste24 [19, 20]. However, at present, the molecular mechanisms that regulate the expression of Zmpste24 have not been fully clarified. Recent studies showed that the gene expression of Zmpste24 was reduced in response to oxidative stress [19, 20]. Here, we can speculate that the induction of GMF in non-brain tissue might cause an oxidative stress-related reduction of Zmpste24 [19, 20], probably due to its vulnerability to oxidative stress [14]. We believe that GMF overexpression in local tissue might contribute to the promotion of the local aging process by causing secondary laminopathy. GMF is ectopically induced by proteinuria in renal tubules [7, 8]. CKD associated with proteinuria might accelerate the regular aging process in kidney tissue and also enhance the progression of CKD, which consequently might have an impact on systemic pathological changes, including organismal aging, cardio-vascular damage and inflammatory changes.

Several *in vivo* studies have demonstrated that senescent cells accumulate with age [22]. Senescence is considered to be related to organismal aging, through the disruption of tissue functions [1, 3, 22, 23]. Senescence is regulated by the p53 or p16-Rb pathway, both of which are activated in the presence of oxidative stress [22, 23]. Some studies have demonstrated that an accumulation of prelamin A induces a significant increase in senescence-associated biomarkers, such as SA-β-gal staining [19, 32]. This suggests that the reduction of Zmpste24 levels and the accumulation of prelamin A might be linked to the activation of the p53 pathway [32], leading to senescence and premature aging [22, 23]. On the other hand, Kudlow et al. reported that the expression of p53 target genes was not highly up-regulated in laminopathy cases [35]. Varela et al. examined whether or not the absence of p53 could result in a recovery of the premature-aging phenotypes that were observed in the laminopathy model mice [32]. Zmpste24−/− p53−/− mice exhibit a gain in weight and an increased lifespan, compared with Zmpste24−/− mice [32]. The expression of p53 target genes, such as the *p21/waf1* gene, decreased in Zmpste24−/− p53−/− mice, compared with age-matched Zmpste24−/− mice [32]. In Zmpste24−/− p53−/− mice, the phenotypes seen in the Zmpste24−/− mice were partially improved by the absence of the *p53* gene [32]. These findings indicate that the activation of the p53 pathway might play a role in the progression of aging in laminopathy cases.

In the present study, the expression of p21/waf1 mRNA and protein increased in the GMF-TG mice at 10 weeks (Figure 9A and C), suggesting that premature aging in the young GMF-TG mice might be associated with the p53 pathway. To our surprise, the GMF-TG mice showed a reduced expression of p21/waf1 mRNA at 60 weeks (Figure 9B). There was no change in the expression of the p21/waf1 protein (Figure 9C). Because the p21/waf1 is a key mediator of the p53-dependent cell cycle arrest and senescence process, the expression of p21/waf1 is exquisitely regulated by transcriptional, post-transcriptional and post-translational mechanisms [36]. We can speculate that the regulatory factors of the p21/waf1 might be influential in the old GMF-TG mice in order to modulate p53-dependent senescence. For example, the p400 E1A-associated protein, which inhibits p53-dependent p21/waf1 transcription [37], modulates cell fate decisions (cell cycle progression, apoptosis, or senescence) by the regulation of ROS homeostasis [38]. It is conceivable that the up-regulation of p400 might be induced in the later age of GMF-TG mice in order to modulate increased oxidative stress caused by the overexpressed GMF in non-brain tissue. Further studies are required to elucidate these issues.

**Figure 10:**
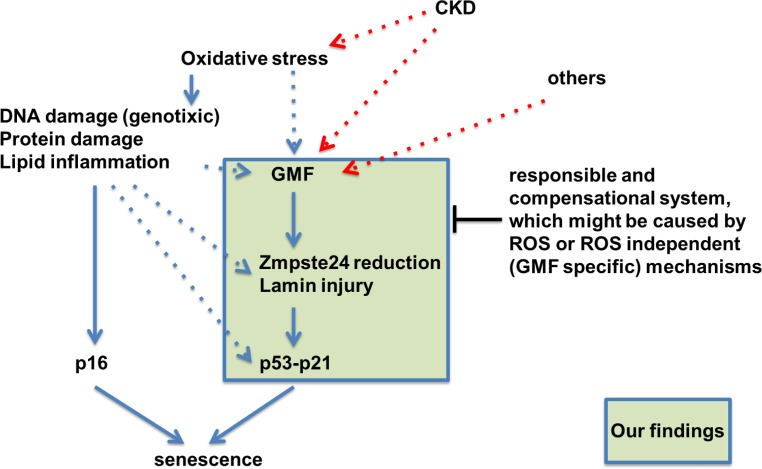
Hypothetical schematic representation of the novel role of GMF overexpression in non-brain tissue *in vivo* This study showed that the GMF-TG mice developed accelerated aging phenotypes due to lamin injury caused by oxidative stress-related reduction of Zmpste24. Zmpste24 is down-regulated in response to oxidative stress [19, 20], which appears to be associated with the activation of p53 and p16-Rb pathways [19]. Oxidative stress activates the p53 and p16-Rb pathways, inducing senescence and eventually leading to accelerated aging [22, 23]. It is conceivable that the ectopic GMF overexpression might be a factor connecting aging and lamin injury through oxidative stress.

In conclusion, this study demonstrated that the GMF-TG mice showed mild accelerated aging phenotypes, as a consequence of secondary laminopathy. In regard to the mechanisms involved in this process, we propose that the ectopic GMF overexpression induced an oxidative stress-related reduction of Zmpste24. This might be associated with the activation of the signaling-pathway via the disassembly of nuclear envelope proteins, leading to senescence in cells and tissue [39]. On the basis of the findings reported here, we propose a hypothetical, novel role of GMF overexpression in non-brain tissue *in vivo* (Figure 10). Much attention has been paid to a functional link between lamin A and the physiological aging process [17–20]. It is also well known that enhanced oxidative stress has been implicated in aging, and age-related diseases, such as cardiovascular disease, cancer, neurodegenerative diseases, and other chronic conditions [22, 23, 40]. Our findings suggest the possibility that GMF-TG mice might provide a novel premature-aging model useful in clarifying the relationship between aging, the pathophysiology of laminopathies and the clinical relevance for various chronic disease states.

## MATERIALS AND METHODS

### Ethics statement

All of the animal experiments employed in this study were conducted in accordance with protocols approved by the Ethical Committee of Kobe Women's University on Animal Research (Permit Number: 154, 181, 209). All efforts possible were made to minimize animal suffering.

### Production of transgenic mice overexpressing GMF

First, we prepared a construct to create transgenic mice overexpressing GMF (GMF-TG mice) by cloning the coding region of GMF (97–915 bp) to a pCAGGS vector [41]. The purified construct was used for the microinjection of fertilized oocytes from C57BL6/J mice. The transgenic mice were established at Oriental BioService (Kyoto, Japan) and maintained on a C57BL6/J genetic background. GMF-TG mice were identified by PCR screening.

### Animals and diets

C57BL6/J (wild-type) mice were purchased from Crea Japan Inc. (Tokyo, Japan). GMF-TG and wild-type male mice were used for the analyses. All of the mice employed in the study were housed in standard cages with 3–4 mice per cage under controlled temperature (21 ± 2°C) and humidity (50%) conditions, with a 12 h light/dark cycle. The mice were given free access to tap water and basal diets (CE-2, Crea Japan Inc., Tokyo, Japan) throughout the experiments.

The mice were kept until natural death and monitored twice a week for clinical signs, morbidity or mortality during the experimental period of 155 weeks. The clinical signs used to assess health and welfare were characterized by obvious symptoms, like the moribund state, hypokinesia, fever, severe cachexia, loss of body weight, lack of grooming or nesting, not eating or drinking, alopecia, skin atrophy and spinal curvature [21, 42]. After fasting overnight, euthanasia was performed by an inhaled anesthetic overdose, followed by sevoflurane (Sevofrane, Maruishi Pharma., Osaka, Japan) in order to minimize animal suffering from distress or pain.

Histological analyses, PCR analyses and Western blot analyses: Tissue samples were obtained after the euthanasia.

PCR screening, hair-growth assay and creatinine levels: The experiments were performed under anesthesia maintained by the inhalation of sevoflurane.

### PCR screening

Genomic DNA was extracted from 1–2 mm sections of the tail tip. The DNA was purified using the Wizard SV Genomic DNA Purification System (Promega, Madison, WI), according to the manufacturer's instructions. The PCR screening was carried out using the T3 (5′-AATTAACCCTCACTAA AGGG-3′) and T7 (5′-GTAATACGACTCACTATAGG GC-3′) primer sequences and TaKaRa Taq (TaKaRa, Shiga, Japan) under the following conditions: 94°C 90 s (1 cycle); 94°C 60 s, 55°C 60 s, 72°C 90 s (35 cycles) in the GeneAmp PCR System 9700 (Life technologies, Carlsbad, CA).

### Histological analyses

Mice were sacrificed at 30 weeks for the histological analyses. Tissue sections were fixed in 10% neutral-buffered formalin solution, paraffin embedded, sectioned, and stained with hematoxylin-eosin (HE), periodic acid–schiff (PAS) and masson trichrome (MT), all of which were performed at Applied Medical Research (Osaka, Japan). The sections were examined using an Olympus BX51 microscope & DP70 digital camera system (Olympus, Tokyo, Japan).

### Hair-growth assay

Employing age-matched mice, dorsal hair was removed by depilatory cream from a square grid of skin measuring 1.5 cm×1.5 cm. Hair regrowth was scored 15 days later from digital photographs and a semi-quantitative assessment was done using 6–9 hair samples/animal. A square measuring 0.5 cm×0.5 cm was also used for the collection of hair samples.

### Creatinine levels

When the mice were 60–80 weeks old, blood samples were collected by venipuncture from the caudal vein into syringes without anticoagulant (for serum samples). Serum creatinine levels were measured employing the CRE-EN kit (Kainos, Tokyo, Japan), according to the manufacturer's instructions.

### RNA extraction and reverse transcription for PCR analyses

Total mouse tissue RNA was extracted with TRIzol reagent (Life technologies), according to the manufacturer's instructions, followed by DNase treatment to eliminate contaminating genomic DNA. Single-strand DNA was generated from the RNA with random hexamers primers using the Transcriptor First Strand cDNA synthesis kit (Roche, Mannheim, Germany).

### Confirmation of gene expression of GMF

Quantitative PCR analyses of the GMF genes were performed with TaqMan Universal PCR Master Mix (Life technologies) and TaqMan probe of GMF-β and mouse GAPDH in the 7500 Fast Real-Time PCR System (Life technologies) The TaqMan probes employed were obtained from Life technologies. The expression levels of the GMF mRNA were normalized to those of the GAPDH mRNA.

### Gene expression of Zmpste24, TGF-β1, CTGF, and p21/waf1

Real-time PCR analyses were performed with SYBR Premix Reagent (TaKaRa) in the 7500 Fast Real-Time PCR System (Life technologies). The following primer pairs were used for real-time PCR analyses: The GAPDH forward primer sequence 5′-AAATGGTGAAGGTCGGTGTG-3′, and its reverse primer sequence 5′-TGAAGGGGTCGTTGATGG-3′, The Zmpste24 forward primer sequence 5′-CCTTCAGCTTCTGGTCAGGACTCTA-3′, and its reverse primer sequence 5′-CTGGTCCAAAGCCAGCAGAAC-3′, The TGF-β1 forward primer sequence 5′-GTGTGGAGCAACATGTGGAACTCTA-3′, and its reverse primer sequence 5′-TTGGTTCAGCCACTGCCGTA-3′, The CTGF forward primer sequence 5′-ACCCGAGTTACCAATGACAATACC-3′, and its reverse primer sequence 5′-CCGCAGAACTTAGCCCTGTATG-3′, The p21/waf1 forward primer sequence 5′-CTGTCTTGCACTCTGGTGTCTCA-3′, and its reverse primer sequence 5′-CCAATCTGCGCTTGGAGTGA-3′. The expression level of each mRNA was normalized to that of the corresponding GAPDH mRNA.

### Western blot analyses

The total protein obtained from each mouse kidney was extracted with RIPA buffer [43], and protease inhibitor (Roche). A total of 50 μg of each sample was separated by 5–20% polyacrylamide gel and transferred to a nylon membrane (Hybond-P: GE Healthcare, Buckinghamshire, UK). Blots were blocked with 5% ECL Blocking Agent (GE Healthcare) in TBS plus 0.1% Tween20 (Sigma-Aldrich, St. Louis, MO), and incubated overnight at 4°C with 1/400 Lamin A/C polyclonal antibody rabbit (#3267–100, Bio Vision, Milpitas, CA), 1/1,000 p21 monoclonal antibody mouse (#60214–1-Ig, Proteintech, Chicago, IL), or 1/4000 alpha actin polyclonal antibody rabbit (#23660–1-AP, Proteintech). Finally, the blots were incubated with 1/10,000 HRP-linked anti-rabbit IgG or anti-mouse IgG (GE Healthcare). Antibody binding was detected with the ECL Prime chemiluminescence system (GE Healthcare), with subsequent exposure to LAS-3000 chemiluminescence (GE Healthcare). The protein expression of p21/waf1 was assayed employing the western blot method and quantified using Image J densitometry software.

### Statistical analyses

The data analyses were performed using the Kaleida Graph software package (Synergy Software, Tokyo, Japan). Values were expressed as means ± S.E. Statistical analyses for the comparison of two groups were performed using Unpaired Student's t test. For the lifespan assessments, data were analyzed employing the Kaplan-Meier method and log-rank test using StatMate'3 software package (ATMS Inc., Tokyo, Japan). P values < 0.05 were considered to indicate statistical significance.

## SUPPLEMENTAL DATA TABLE AND FIGURES



## References

[1] Vijg J, Campisi J (2008). Puzzles, promises and a cure for ageing. Nature.

[2] Baker DJ, Wijshake T, Tchkonia T, LeBrasseur NK, Childs BG, van de Sluis B, Kirkland JL, van Deursen JM (2011). Clearance of p16Ink4a-positive senescent cells delays ageing-associated disorders. Nature.

[3] Sahin E, Depinho RA (2010). Linking functional decline of telomeres, mitochondria and stem cells during ageing. Nature.

[4] Coresh J, Selvin E, Stevens LA, Manzi J, Kusek JW, Eggers P, Van Lente F, Levey AS (2007). Prevalence of chronic kidney disease in the United States. JAMA.

[5] Liew G, Mitchell P, Wong TY, Iyengar SK, Wang JJ (2008). CKD increases the risk of age-related macular degeneration. J Am Soc Nephrol.

[6] Ruggenenti P, Perna A, Mosconi L, Pisoni R, Remuzzi G (1998). Urinary protein excretion rate is the best independent predictor of ESRF in non-diabetic proteinuric chronic nephropathies “Gruppo Italiano di Studi Epidemiologici in Nefrologia” (GISEN). Kidney Int.

[7] Takenaka M, Imai E, Kaneko T, Ito T, Moriyama T, Yamauchi A, Hori M, Kawamoto S, Okubo K (1998). Isolation of genes identified in mouse renal proximal tubule by comparing different gene expression profiles. Kidney Int.

[8] Nakajima H, Takenaka M, Kaimori JY, Nagasawa Y, Kosugi A, Kawamoto S, Imai E, Hori M, Okubo K (2002). Gene expression profile of renal proximal tubules regulated by proteinuria. Kidney Int.

[9] Lim R, Zaheer A (1996). In vitro enhancement of p38 mitogen-activated protein kinase activity by phosphorylated glia maturation factor. J Biol Chem.

[10] Zaheer A, Zaheer S, Sahu SK, Knight S, Khosravi H, Mathur SN, Lim R (2007). A novel role of glia maturation factor: induction of granulocyte-macrophage colony-stimulating factor and pro-inflammatory cytokines. J Neurochem.

[11] Kempuraj D, Khan MM, Thangavel R, Xiong Z, Yang E, Zaheer A (2013). Glia maturation factor induces interleukin-33 release from astrocytes: implications for neurodegenerative diseases. J Neuroimmune Pharmacol.

[12] Thangavel R, Kempuraj D, Stolmeier D, Anantharam P, Khan M, Zaheer A (2013). Glia maturation factor expression in entorhinal cortex of Alzheimer's disease brain. Neurochem Res.

[13] Stolmeier D, Thangavel R, Anantharam P, Khan MM, Kempuraj D, Zaheer A (2013). Glia maturation factor expression in hippocampus of human Alzheimer's disease. Neurochem Res.

[14] Kaimori JY, Takenaka M, Nakajima H, Hamano T, Horio M, Sugaya T, Ito T, Hori M, Okubo K, Imai E (2003). Induction of glia maturation factor-beta in proximal tubular cells leads to vulnerability to oxidative injury through the p38 pathway and changes in antioxidant enzyme activities. J Biol Chem.

[15] Zaheer A, Yang B, Cao X, Lim R (2004). Decreased copper-zinc superoxide dismutase activity and increased resistance to oxidative stress in glia maturation factor-null astrocytes. Neurochem Res.

[16] Broers JL, Ramaekers FC, Bonne G, Yaou RB, Hutchison CJ (2006). Nuclear lamins: laminopathies and their role in premature ageing. Physiol Rev.

[17] Scaffidi P, Misteli T (2006). Lamin A-dependent nuclear defects in human aging. Science.

[18] McClintock D, Ratner D, Lokuge M, Owens DM, Gordon LB, Collins FS, Djabali K (2007). The mutant form of lamin A that causes Hutchinson-Gilford progeria is a biomarker of cellular aging in human skin. PloS one.

[19] Ragnauth CD, Warren DT, Liu Y, McNair R, Tajsic T, Figg N, Shroff R, Skepper J, Shanahan CM (2010). Prelamin A acts to accelerate smooth muscle cell senescence and is a novel biomarker of human vascular aging. Circulation.

[20] Lattanzi G, Ortolani M, Columbaro M, Prencipe S, Mattioli E, Lanzarini C, Maraldi NM, Cenni V, Garagnani P, Salvioli S, Storci G, Bonafe M, Capanni C (2014). Centenarian lamins: rapamycin targets in longevity. J Cell Sci.

[21] Takeda T, Hosokawa M, Takeshita S, Irino M, Higuchi K, Matsushita T, Tomita Y, Yasuhira K, Hamamoto H, Shimizu K, Ishii M, Yamamuro T (1981). A new murine model of accelerated senescence. Mech Ageing Dev.

[22] Chen JH, Hales CN, Ozanne SE (2007). DNA damage, cellular senescence and organismal ageing: causal or correlative?. Nucleic Acids Res.

[23] Pelicci PG (2004). Do tumor-suppressive mechanisms contribute to organism aging by inducing stem cell senescence?. J Clin Invest.

[24] Mounkes LC, Kozlov S, Hernandez L, Sullivan T, Stewart CL (2003). A progeroid syndrome in mice is caused by defects in A-type lamins. Nature.

[25] Tyner SD, Venkatachalam S, Choi J, Jones S, Ghebranious N, Igelmann H, Lu X, Soron G, Cooper B, Brayton C, Hee Park S, Thompson T, Karsenty G (2002). p53 mutant mice that display early ageing-associated phenotypes. Nature.

[26] Kondratov RV, Kondratova AA, Gorbacheva VY, Vykhovanets OV, Antoch MP (2006). Early aging and age-related pathologies in mice deficient in BMAL1, the core componentof the circadian clock. Genes Dev.

[27] Li L, Zhao D, Wei H, Yao L, Dang Y, Amjad A, Xu J, Liu J, Guo L, Li D, Li Z, Zuo D, Zhang Y (2013). REGgamma deficiency promotes premature aging via the casein kinase 1 pathway. Proc Natl Acad Sci U S A.

[28] Lee YF, Liu S, Liu NC, Wang RS, Chen LM, Lin WJ, Ting HJ, Ho HC, Li G, Puzas EJ, Wu Q, Chang C (2011). Premature aging with impaired oxidative stress defense in mice lacking TR4. American journal of physiology Endocrinology and metabolism.

[29] Merideth MA, Gordon LB, Clauss S, Sachdev V, Smith AC, Perry MB, Brewer CC, Zalewski C, Kim HJ, Solomon B, Brooks BP, Gerber LH, Turner ML (2008). Phenotype and course of Hutchinson-Gilford progeria syndrome. N Engl J Med.

[30] Ruiz-Torres MP, Bosch RJ, O'Valle F, Del Moral RG, Ramirez C, Masseroli M, Perez-Caballero C, Iglesias MC, Rodriguez-Puyol M, Rodriguez-Puyol D (1998). Age-related increase in expression of TGF-beta1 in the rat kidney: relationship to morphologic changes. J Am Soc Nephrol.

[31] Lim JH, Kim EN, Kim MY, Chung S, Shin SJ, Kim HW, Yang CW, Kim YS, Chang YS, Park CW, Choi BS (2012). Age-associated molecular changes in the kidney in aged mice. Oxid Med Cell Longev.

[32] Varela I, Cadinanos J, Pendas AM, Gutierrez-Fernandez A, Folgueras AR, Sanchez LM, Zhou Z, Rodriguez FJ, Stewart CL, Vega JA, Tryggvason K, Freije JM, Lopez-Otin C (2005). Accelerated ageing in mice deficient in Zmpste24 protease is linked to p53 signalling activation. Nature.

[33] Zhang H, Kieckhaefer JE, Cao K (2013). Mouse models of laminopathies. Aging cell.

[34] Fong LG, Ng JK, Meta M, Cote N, Yang SH, Stewart CL, Sullivan T, Burghardt A, Majumdar S, Reue K, Bergo MO, Young SG (2004). Heterozygosity for Lmna deficiency eliminates the progeria-like phenotypes in Zmpste24-deficient mice. Proc Natl Acad Sci U S A.

[35] udlow BA, Stanfel MN, Burtner CR, Johnston ED, Kennedy BK (2008). Suppression of proliferative defects associated with processing-defective lamin A mutants by hTERT or inactivation of p53. Mol Biol Cell.

[36] Sullivan KD, Gallant-Behm CL, Henry RE, Fraikin JL, Espinosa JM (2012). The p53 circuit board. Biochim Biophys Acta.

[37] Chan HM, Narita M, Lowe SW, Livingston DM (2005). The p400 E1A-associated protein is a novel component of the p53 --> p21 senescence pathway. Genes Dev.

[38] Mattera L, Courilleau C, Legube G, Ueda T, Fukunaga R, Chevillard-Briet M, Canitrot Y, Escaffit F, Trouche D (2010). The E1A-associated p400 protein modulates cell fate decisions by the regulation of ROS homeostasis. PLoS Genet.

[39] Sieprath T, Darwiche R, De Vos WH (2012). Lamins as mediators of oxidative stress. Biochem Biophys Res Commun.

[40] Rahman K (2007). Studies on free radicals, antioxidants, and co-factors. Clin Interv Aging.

[41] Niwa H, Yamamura K, Miyazaki J (1991). Efficient selection for high-expression transfectants with a novel eukaryotic vector. Gene.

[42] Franco NH, Correia-Neves M, Olsson IA (2012). Animal welfare in studies on murine tuberculosis: assessing progress over a 12-year period and the need for further improvement. PloS one.

[43] Li AL, Li HY, Jin BF, Ye QN, Zhou T, Yu XD, Pan X, Man JH, He K, Yu M, Hu MR, Wang J, Yang SC (2004). A novel eIF5A complex functions as a regulator of p53 and p53-dependent apoptosis. J Biol Chem.

