# Supplementary material for: Transgenic mice overexpressing glia maturation factor-β, an oxidative stress inducible gene, show premature aging due to Zmpste24 down-regulation
Source: Aging (Albany NY). 2015 Jul 13;7(7):486–98. doi: 10.18632/aging.100779 (PMC4543038; doi:10.18632/aging.100779)
Supplement: Supplementary file 1 [file aging-07-486-s001.pdf]

SUPPLEMENTAL DATA

Supplemental Table 1. Phenotypes seen in the appearance of WT and GMF-TG mice during the experimental period of 155 weeks.

	alopecia	skin atrophy	spinal curvature
GMF-TG (n=97)	n=17 (18%)	n=9 (9%)	n=9 (9%)
WT (n=41)	n=6 (15%)	n=0 (0%)	n=0 (0%)

There was no detectable differences in body size, jaws or limbs between the WT and GMF-TG mice during the study.

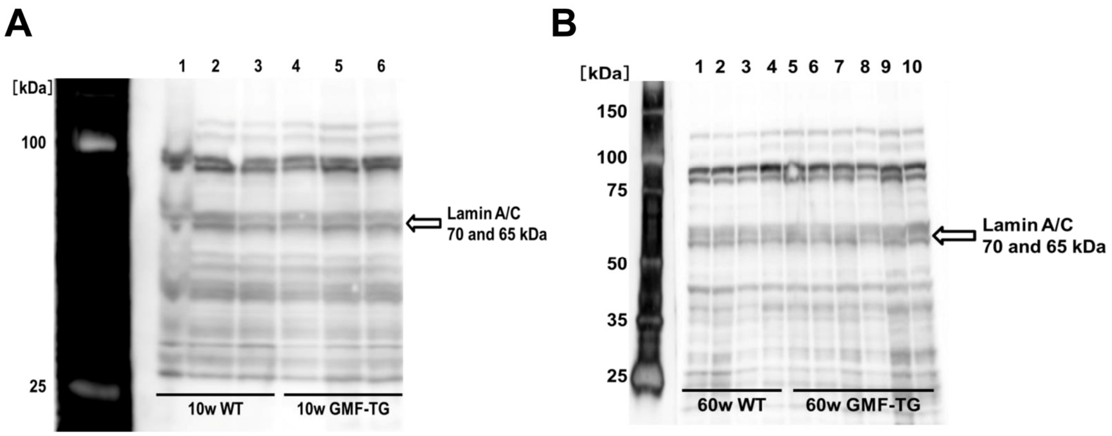

Supplemental Figure 1. Western blot of lamin A/C in WT and GMF-TG mice. (**A**) This figure shows the results from western blot analyses of lamin A/C at 10 weeks. Figure 6A shows the results from lanes 2-6. (**B**) This figure shows the results from western blot analyses of lamin A/C at 60 weeks. Figure 6B shows the results from lanes 1-3 and 5-8.

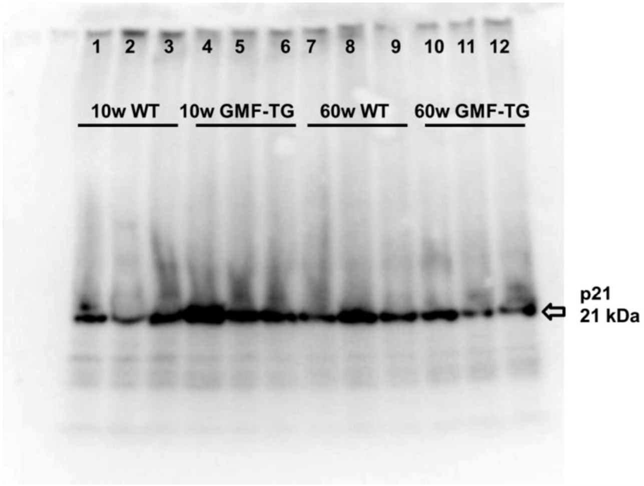

Supplemental Figure 2. Western blot of p21/waf1 in WT and GMF-TG mice. This figure shows the results from western blot analyses of p21/waf1 at 10 and 60 weeks. Figure 9C shows the results from lanes 1-12.
